# Patients' Knowledge and Information Needs about Isotretinoin Therapy Use in Jordan

**DOI:** 10.1155/2022/9443884

**Published:** 2022-02-14

**Authors:** Anan S. Jarab, Sayer Al-Azzam, Shriefa Almutairi, Tareq L. Mukattash

**Affiliations:** Department of Clinical Pharmacy, Faculty of Pharmacy, Jordan University of Science and Technology, P.O. Box 3030, Irbid 22110, Jordan

## Abstract

**Background:**

Despite being the first-line treatment for severe or moderate acne, isotretinoin has several serious side effects that necessitate the evaluation of patients' knowledge about isotretinoin side effects and its proper use.

**Objective:**

The current study aim was to explore information needs about isotretinoin by evaluating patients' knowledge about the appropriate use of isotretinoin and its associated side effects.

**Methods:**

In addition to the sociodemographic variables, a validated online questionnaire was adopted from the literature to evaluate patients' knowledge about isotretinoin use and its potential side effects. Independent *t*-test and one-way analysis of variance (ANOVA) test were implemented to find the correlation between the study variables and the knowledge score.

**Results:**

The most recognized side effect of isotretinoin therapy was dryness (98.1%). The study patients showed good knowledge about isotretinoin use with a mean knowledge score of 8.1 (SD = 0.7). However, more than half of them (61.0%) mistakenly thought that isotretinoin therapy should be taken continuously for more than 6 months without stop, and some of them did not know that isotretinoin is recommended to be taken with fatty meal (24%) and sunblock (24.6%). Female gender (8.2 (SD = 0.8)) and using isotretinoin for more than 6 months (8.3 (SD = 1.2)) were significantly associated with a higher knowledge score of isotretinoin use (*p*=0.01), when compared with male patients (7.8 (SD = 0.7)) and less than 6-month use of isotretinoin (7.7 (SD = 0.7)).

**Conclusions:**

The lack of patients' information about the potential side effects, duration of therapy, and some instructions on isotretinoin use, such as taking the medication with fatty meal and sunblock, shed the light on the necessity to prepare leaflets, educational brochures, and educational posts via social media in order to improve patients' knowledge about isotretinoin therapy and its optimal use.

## 1. Introduction

As stated by the Global Burden of Disease study, acne vulgaris (AV) affects about 85% of individuals between the ages of 12 and 25 years, and its effect extends to younger children [1, 2]. Isotretinoin is approved as first-line therapy for the treatment of severe or moderate acne that does not respond to other medications [2–4]. Nevertheless, there are many side effects of isotretinoin that range from mild to life-threatening [5, 6]. The clinical adverse effects of isotretinoin can be categorized into mucocutaneous such as cheilitis and facial dermatitis and systemic such as teratogenicity [2–4]. Other potential side effects include dryness, dyslipidemia, nosebleeds, dizziness, eye inflammation, joint and back pain, depression, and abnormal liver function [7]. Congenital defects are estimated to occur in up to 35% of infants exposed to the drug in utero, and neurocognitive deficits have been reported to affect 30–60% of children exposed to isotretinoin prenatally [8]. On the contrary, most patients do not have enough knowledge about the potential side effects of isotretinoin and how to deal with them, and there are no specific risk minimization tools other than the warnings in the product leaflet [9], which increase the chance of unwanted adverse effects [6, 10, 11]. A recent Saudi study reported that more than half of the patients did not recognize hyperlipidemia as a side effect of isotretinoin. Furthermore, more than one-third of them did not know that isotretinoin can elevate liver enzymes' level [9]. Another study conducted in Saudi Arabia reported that patients who were prescribed isotretinoin were not sufficiently aware of its proper use [12]. Nearly half of the patients in the later study did not know that they should not donate blood while using isotretinoin. In addition, the majority of the surveyed women did not know that they must stop taking isotretinoin at least one month before becoming pregnant [12]. A recent study showed that patients' knowledge about the importance of using contraceptives in women of reproductive age while on isotretinoin is suboptimal [13], with only few proportions of women adhering to the use of contraception during isotretinoin use [14, 15].

The low safety profile of isotretinoin therapy sheds the light on the necessity to improve patients' awareness about the medication and its optimal use. The current study is the first one which evaluated patients' knowledge about the appropriate use of isotretinoin and its associated side effects in Jordan. Findings of the present study are expected to contribute to a deeper understanding of the current situation in the region, help developing more effective interventions that aim at raising patient's awareness and knowledge about isotretinoin use, and ultimately enhancing safety and health outcomes among isotretinoin users.

### 1.1. Aim of the Study

The aim of this study was to explore the gaps in patients' knowledge about isotretinoin use and its associated side effects.

## 2. Methods

### 2.1. Study Design and Subjects

The current cross-sectional study was conducted in Jordan in the period from January through May 2019. An online questionnaire was distributed using Facebook groups and Twitter. A cover letter that clarified the objectives of the study and the inclusion criteria was attached to the questionnaire. Patients were included in the study if they were 13 years or older, had acne, used isotretinoin as treatment and live in Jordan. The cover letter also informed the patients that their participation is voluntary and the data will be kept confidential. Based on the Raosoft program, a minimum sample of 267 patients was required to produce 90% confidence interval and less than 5% margin of error. To adjust for any dropout, 10% of the sample size was added, giving a total sample size of 294 patients.

### 2.2. Study Instrument

After extensive literature review [9, 16–18], the current study survey was designed. The questionnaire was designed in Arabic language and contained three parts. The first part included information about sociodemographic variables such as age, gender, marital status, education level, profession, daily dose of isotretinoin, and the duration since isotretinoin course initiation. The second part evaluated patients' knowledge about different isotretinoin adverse effects including skin dryness, teratogenicity, dyslipidemia, itching, rash, nosebleeds, joint and back pain, dizziness, eye inflammation, depression, and increased liver enzymes. The third part included nine yes/no questions that evaluated patients' knowledge about isotretinoin use including the need for an authorized prescription, the appropriate duration of isotretinoin use, isotretinoin use instructions such as the necessity to take isotretinoin with water and after fatty meal, the importance of using sunblock with isotretinoin, and the necessity to make regular laboratory checkout in addition to the awareness of isotretinoin use during pregnancy, lactation, and blood donation. A score of one was given for each correct answer, and the scores were summed out of nine. The survey was reviewed by experts in the field including a dermatologist and two professors of pharmacy, and changes were made when deemed appropriate. The study survey was also piloted on twenty patients to ensure for the clarity of the questionnaire. The piloted patients were excluded from the main study.

### 2.3. Ethical Approval

Ethical approval of this study was attained from the Institutional Review Board (IRB) of King Abdullah University Hospital and Jordan University of Science and Technology (JUST). The IRB reference number is 48/118/2018.

### 2.4. Data Analysis

Data were coded and entered into SPSS software program version 22 for statistical analysis. Descriptive statistics were made for quantitative variables using frequencies and percentages for categorical variables and mean and standard deviation for continuous variables. Independent *t*-test was conducted to find the association between the dichotomous predictors and the continuous outcome knowledge or awareness score, while one-way analysis of variance (ANOVA) test was used to find the correlation between the categorical predictors of three or more categories and the knowledge score.

## 3. Results

A total of 367 patients completed the online survey. Most of the participants were female (89.1%), in the age group of 16 to 25 years (87.4%), not married (94%), had high education level (93.8%), were students (63%), were receiving 10–20 mg isotretinoin daily (85.2%), and they were taking isotretinoin for less than 6 months (55.3%). Demographic characteristics of the study participants are presented in Table 1.

The majority of the patients (67.6%) knew about isotretinoin therapy from their physicians, while the rest of the patients knew about this therapy from other sources including pharmacists, friends, family members, and internet resources. Most of the patients (93.2%) advised others to use isotretinoin based on their old experience.

The most recognized side effect of isotretinoin therapy was dryness (98.1%), followed by teratogenicity (66.5%) and dyslipidemia (58.9%). Other side effects including itching, rash, nosebleeds, joint and back pain, dizziness, eye inflammation, depression, and increased liver enzymes were recognized by less than half of the patients. Among the seventeen married female patients who participated in this study, only one patient used isotretinoin without oral contraceptives, two patients were not informed by the physician or the pharmacist about the risk of teratogenicity of isotretinoin, and three of them did not make pregnancy test on a monthly basis.

The study patients showed good knowledge about isotretinoin use with a mean total knowledge score of 8.1 (SD = 0.7). As shown in Table 2, more than half of the patients (61.0%) mistakenly thought that isotretinoin therapy should be taken continuously for more than 6 months without stop, and nearly one-fourth of them did not know that isotretinoin is recommended to be taken with fatty meal (24%) and sunblock (24.6%).

As shown in Table 3, the *t*-test reported that female gender (8.2 (SD = 0.8)) and more than 6-month use of isotretinoin (8.3 (SD = 1.2)) were significantly associated with a higher knowledge score of isotretinoin use (*p*=0.01), when compared with male patients (7.8 (SD = 0.7)) and less than 6-month use of isotretinoin (7.7 (SD = 0.7)).

Results showed that only 1.4% of the patients were asked by their physicians to sign a consent form before starting isotretinoin therapy. However, nearly 84.5% of the patients were informed by their physicians about the expected side effect of isotretinoin and how to manage it. Most of the patients (63.5%) made a monthly follow-up with their physicians, 17.2% made a follow-up every two months, and 12.0% made a follow-up every three months or more. Approximately one-third of the patients (34.4%) did not inform their physicians before using other drugs with isotretinoin.

## 4. Discussion

In 1971, clinical trials' results revealed that isotretinoin was ineffective for treating skin cancer, but could be useful for the management of AV. However, due to concerns about its side effects and its potential to cause birth defects in particular, the medication was not released to the market until 1982 [19]. Isotretinoin is, by far, the most cost-effective drug when compared with other medications used for severe acne treatment. However, it has been associated with some serious and teratogenic adverse effects that patients must be aware of [2–4]. Therefore, the current study, which is the first one in Jordan, was conducted to evaluate patients' knowledge about isotretinoin use and its accompanying adverse effects.

The majority of this study patients (67.6%) heard about isotretinoin therapy from their physicians. Similar results were reported by Kara Polat (64.4%) [20] and Al-Harbi (61.9%) [10], but higher percentages were reported in studies conducted by Younis and Al-Harbi (89.6%) [9], Bakheet et al. (93%) [21], and Imam et al. (81.9%) [12]. On the contrary, an earlier Saudi study [22] reported that the most identified source of information for isotretinoin therapy was friends, followed by the internet and social media. However, most of the current study patients advised others to use isotretinoin based on their old experience, which might increase the potential of medication side effects.

Consistent with earlier study findings [9, 12, 20–22], dryness was the most commonly recognized side effect of isotretinoin therapy. The majority of the current study participants were not able to recognize side effects of isotretinoin therapy such as itching, rash, nosebleeds, joint and back pain, dizziness, eye inflammation, depression, and increased liver enzymes. In comparison, only 28.6% and 33.5% of the patients participated in a Saudi study recognized depression and abnormal liver function as side effects of isotretinoin, respectively [22]. In addition, around half of the isotretinoin users enrolled in another study were not able to recognize depression as a side effect of isotretinoin therapy [21]. Comparable with earlier Saudi study finding [21], some patients were not able to recognize dyslipidemia as a side effect of isotretinoin therapy in the current study. Although the majority of the present study patients recognized teratogenicity as a side effect, higher percentages were reported in earlier studies [9, 12]. Lack of knowledge about isotretinoin side effects in this study could be attributed to the availability of isotretinoin without prescription, which could lead to the missing out of physician explanation about isotretinoin side effects and might make the patients feel that the drug is safe. Furthermore, inadequate community pharmacists' knowledge regarding isotretinoin toxicity may have an impact, given the vital role which pharmacists play as medication experts in providing the information needed about isotretinoin to the patients [22].

Given the teratogenicity of isotretinoin and the fact that 50% of isotretinoin users are women of reproductive age [3, 23], precautious interventions must be implemented while using isotretinoin among these patients. In other words, married female patients who are prescribed isotretinoin must perform pregnancy test before and during the treatment course in order to avoid birth defects [24]. Consistent with earlier Saudi and Canadian studies [25, 26], some married female patients in the present study were not informed by the physician or the pharmacist about the teratogenicity of isotretinoin and did not perform pregnancy test on a monthly basis while using the drug. These findings shed the light on the necessity to implement patient counseling and educational programs which improve patients' information about the potential teratogenic effect and other potential side effects of isotretinoin therapy.

The current study patients' knowledge about isotretinoin use was similar to earlier studies' findings [21, 22] and better than other studies' reports [10, 11, 20]. In the present study, the patients demonstrated poor knowledge about the appropriate duration of isotretinoin therapy and its combination with fatty meal and sunblock. In order to achieve the optimal therapeutic response to isotretinoin therapy, it should be taken for four to six months depending on the daily dose given [27]. More than half of the current study patients mistakenly thought that isotretinoin should be taken continuously for more than six months without stop. A similar finding was reported in two studies that were conducted in Saudi Arabia [9, 21]. In contrast, a study conducted in another region of Saudi Arabia reported that the majority of the patients used isotretinoin for less than six months [12]. Moreover, patients who used isotretinoin for more than six months in the present study were found to have significantly better knowledge about isotretinoin therapy. It seems that the increased experience associated with longer duration of isotretinoin use could justify this finding. Lastly, female patients significantly reported a higher knowledge score than male patients in the present study, which could be justified by the fact that females are more caring for skin and more interested in cosmetics and beauty products when compared to males.

Results of the present study showed that only 1.4% of the patients signed up a consent form before starting isotretinoin therapy. Comparable findings were reported in a Canadian study, where only 13% of the patients were requested to sign a consent form [25]. Emphasis should be placed on improving physicians' knowledge about the recent isotretinoin dispensing protocol to combine a consent form with isotretinoin therapy. Results also revealed that more than one-third of the patients did not perform follow-up with their physicians on a monthly basis and did not inform them before using other drugs concurrently with isotretinoin. Therefore, a regular monitoring and medication review should be considered during the course of isotretinoin therapy.

In summary, healthcare providers should provide patient counseling, along with regular monitoring, to improve patients' knowledge and awareness about the appropriate use of isotretinoin and its potential side effects, in order to enhance patients' safety, prevent isotretinoin-associated harms, and ultimately optimize treatment outcomes among isotretinoin users.

### 4.1. Limitations

A larger sample size would make the findings of this study more robust. The use of self-reported questionnaire could make the respondent misunderstand the question which may cause bias to the study findings.

## 5. Conclusion

The current study demonstrates a margin for improvement in patients' knowledge about isotretinoin and its use. Therefore, it is essential to use educational brochures, medication leaflets, and educational posts on social media which aim at improving patients' knowledge on isotretinoin therapy and its associated side effects, particularly for male patients and those with less duration of isotretinoin use.

## Figures and Tables

**Table 1:** Demographic characteristics of the participating patients (*n* = 367).

Characteristics	*N* (%)
Gender	
Male	40 (10.9%)
Female	327 (89.1%)
Age (years)	
16–25	321 (87.4%)
26–35	37 (10.1%)
36–45	5 (1.4%)
≥46	4 (1.1%)
Marital status	
Married	22 (6%)
Others^†^	345 (94%)
Education level	
Low education^‡^	23 (6.2%)
High education	344 (93.8%)
Occupation	
Unemployed	17 (4.6%)
Student	231 (63%)
Medical field	98 (26.7%)
Nonmedical field	21 (5.7%)
Daily dose of isotretinoin	
10–20 mg	313 (85.2%)
20–40 mg	50 (13.7%)
40–60 mg	4 (1.1%)
Period since starting the current isotretinoin course (months)	
Less than or equal to six	203 (55.3%)
More than six	164 (44.7%)

^†^Single, separated, divorced, and widowed. ^‡^Low education: less than the university level.

**Table 2:** Patients' knowledge about isotretinoin use.

Knowledge item	Yes	No
Isotretinoin can be used without prescription^†^	23 (6.3%)	344 (93.7%)
Isotretinoin can be used for more than 6 months without stop^†^	224 (61.0%)	143 (39.0%)
No need to have laboratory monitoring during isotretinoin use^†^	16 (4.4%)	351 (95.6%)
Isotretinoin should be given with plenty of water to avoid dryness	363 (98.9%)	4 (1.1%)
Isotretinoin is recommended to be given with fatty meal	279 (76.0%)	88 (24.0%)
Isotretinoin is recommended to be used with sunblock	277 (75.4%)	90 (24.6%)
Isotretinoin can be used during pregnancy^†^	8 (2.1%)	359 (97.9%)
Blood donation is allowed during isotretinoin therapy^†^	6 (1.6%)	361 (98.4%)
Lactation is allowed during isotretinoin therapy^†^	3 (0.8%)	364 (99.2%)

^†^The correct answer is no.

**Table 3:** Association between sociodemographics and the total knowledge score.

Characteristics	Mean (SD)^§^	*p* value
Gender		
Male	7.8 (0.7)	0.01^*∗*^
Female	8.2 (0.8)	
Marital status		
Married	8.2 (0.6)	0.92
Others^†^	8.1 (0.8)	
Age		
16–25	8.2 (0.8)	0.65
26–35	8.1 (0.8)	
36–45	7.9 (0.7)	
≥46	7.7 (0.5)	
Education level		
Low education^‡^	8.6 (0.7)	0.41
High education	8.0 (0.7)	
Occupation		
Unemployed	8.6 (0.5)	0.85
Student	8.1 (0.8)	
Medical profession	8.2 (0.7)	
Nonmedical profession	8.2 (0.7)	
Duration of usage		
Less than or equal to six	7.7 (0.7)	0.01^*∗*^
More than six	8.3 (1.2)	
Doses		
10–20 mg	8.1 (0.8)	0.43
20–40 mg	8.2 (0.7)	
40–60 mg	7.7 (0.9)	

^§^One-way analysis of variance (ANOVA) and independent *t*-test were applied where applicable ^*∗*^*p* < 0.05 showed a significant difference compared to other groups. ^†^Single, separated, divorced, and widowed. ^‡^Low education: less than the university level.

## Data Availability

The data are available at the corresponding author disk and could be provided upon request.

## Additional Points

*What's Known*. (i) Despite being the first-line treatment of severe or moderate acne, isotretinoin has several serious side effects that necessitate the evaluation of patients' knowledge about isotretinoin side effects and its proper use. (ii) The low safety profile of isotretinoin therapy sheds the light on the necessity to improve patients' awareness about the medication and its optimal use. *What This Study Adds*. (i) The current study revealed lack of patients' information about the potential side effects, duration of isotretinoin use, and some instructions regarding isotretinoin use including taking the medication with fatty meal and sunblock. (ii) Findings of the present study should help exploring the information need about isotretinoin therapy and its use which should be targeted in the future disease management program which aim to enhance safety and health outcomes among isotretinoin users.

## References

[1] Lynn D., Umari T., Dellavalle R., Dunnick C. (2016). The epidemiology of *Acne vulgaris* in late adolescence. *Adolescent Health, Medicine and Therapeutics*.

[2] Strauss J. S., Krowchuk D. P., Leyden J. J. (2007). Guidelines of care for acne vulgaris management. *Journal of the American Academy of Dermatology*.

[3] Azoulay L., Oraichi D., Bérard A. (2006). Patterns and utilization of isotretinoin for acne from 1984 to 2003: is there need for concern?. *European Journal of Clinical Pharmacology*.

[4] Layton A. (2009). The use of isotretinoin in acne. *Dermato-Endocrinology*.

[5] Wilson R. D. (2016). A preventable teratology: isotretinoin. *Canadian Medical Association Journal: Canadian Medical Association Journal*.

[6] Brzezinski P., Borowska K., Chiriac A., Smigielski J. (2017). Adverse effects of isotretinoin: a large, retrospective review. *Dermatologic Therapy*.

[7] https://www.rxlist.com/accutane-drug.htm.

[8] Choi J. S., Koren G., Nulman I. (2013). Pregnancy and isotretinoin therapy. *Canadian Medical Association Journal*.

[9] Younis N. S., Al-Harbi N. Y. (2019). Public understanding and awareness of isotretinoin use and safety in Al Ahsa, Eastern Saudi arabia. *Therapeutic Innovation & Regulatory Science*.

[10] Al-Harbi M. (2010). Concerns and awareness of acne patients about Isotretinoin in Qassim region of Saudi. *International Journal of Health Sciences*.

[11] Jones K. L. (2001). Isotretinoin and pregnancy. *JAMA*.

[12] Imam M. S., Abdel-Sattar R. M., Aldajani G. A., Alsultan R. S., Aldajani Y. A. (2021). Knowledge and awareness of acne patients about isotretinoin use and safety in dawadmi governorate, riyadh region, Saudi Arabia. *Systematic Reviews in Pharmacy*.

[13] Ibrahim A. A. M. (2021). Awareness of isotretinoin use and Saudi FDA pregnancy prevention program in Riyadh, Saudi Arabia: a cross-sectional study among female patients. *Saudi Pharmaceutical Journal*.

[14] Biset N., Lelubre M., Senterre C. (2018). Assessment of medication adherence and responsible use of isotretinoin and contraception through Belgian community pharmacies by using pharmacy refill data. *Patient Preference and Adherence*.

[15] Uusküla A., Pisarev H., Kurvits K., Laius O., Laanpere M., Uusküla M. (2018). Compliance with pregnancy prevention recommendations for isotretinoin in Estonia in 2012–2016. *Drugs—Real World Outcomes*.

[16] Alrwisan A. A., Alshammari T. M., Tahir K. W., Aleissa F. M., Aljadhey H. S. (2014). Community pharmacists’ knowledge of isotretinoin safety. *Saudi Medical Journal*.

[17] Cragan J. D., Friedman J. M., Holmes L. B., Uhl K., Green N. S., Riley L. (2006). Ensuring the safe and effective use of medications during pregnancy: planning and prevention through preconception care. *Maternal and Child Health Journal*.

[18] Lelubre M., Hamdani J., Senterre C. (2018). Evaluation of compliance with isotretinoin PPP recommendations and exploration of reasons for non-compliance: survey among French-speaking health care professionals and patients in Belgium. *Pharmacoepidemiology and Drug Safety*.

[19] Hartmann D., Bollag W. (1993). Historical aspects of the oral use of retinoids in acne. *The Journal of Dermatology*.

[20] Kara Polat A. (2020). Knowledge levels and concerns about oral isotretinoin treatment in the parents of adolescent acne patients. *Dermatologic Therapy*.

[21] Bakheet K. M. A., Alghanemi R. G., Alsiyoufi A. M., Abduljabbar M., Hariri J. (2020). Females’ knowledge and use of isotretinoin (Roaccutane) in the Western region of Saudi arabia. *Cureus*.

[22] Molla A., Alrizqi H., Alruhaili E., Alrizqi S., Alsubhi A. (2020). Assessment of knowledge, attitude, and practice in relation to use of isotretinoin among Al-Madinah population, Saudi Arabia. *International Journal of Medicine in Developing Countries*.

[23] Gollnick H. P., Graupe K., Zaumseil R. P. (2001). Comparison of combined azelaic acid cream plus oral minocycline with oral isotretinoin in severe acne. *European Journal of Dermatology: EJD*.

[24] https://medlineplus.gov/druginfo/meds/a681043.html.

[25] Boucher N., Beaulac-Baillargeon L. (2006). Pregnancy prevention among women taking isotretinoin: failure to comply with the recommendations. *Canadian Family Physician Medecin de Famille Canadien*.

[26] Albadr T., Alruhaimi D., Cahusac P. B., Rohra D. (2019). Knowledge and use of isotretinoin in Saudi female college students: cross-sectional study. *Journal of Dermatology & Dermatologic Surgery*.

[27] Perspectives P. (2015). Treating acne with isotretinoin. *Pediatric Dermatology*.

